# Thigmotropism of Malignant Melanoma Cells

**DOI:** 10.1155/2012/362784

**Published:** 2011-11-17

**Authors:** Pascale Quatresooz, Claudine Piérard-Franchimont, Fanchon Noël, Gérald E. Piérard

**Affiliations:** Department of Dermatopathology, University Hospital of Liège, 4000 Liège, Belgium

## Abstract

During malignant melanoma (MM) progression including incipient metastasis, neoplastic cells follow some specific migration paths inside the skin. In particular, they progress along the dermoepidermal basement membrane, the hair follicles, the sweat gland apparatus, nerves, and the near perivascular space. These features evoke the thigmotropism phenomenon defined as a contact-sensing growth of cells. This process is likely connected to modulation in cell tensegrity (control of the cell shape). These specifically located paucicellular aggregates of MM cells do not appear to be involved in the tumorigenic growth phase, but rather they participate in the so-called “accretive” growth model. These MM cell collections are often part of the primary neoplasm, but they may, however, correspond to MM micrometastases and predict further local overt metastasis spread.

## 1. Introduction

Cancer remains one of the most complex diseases and, despite the impressive advances made in molecular and cell biology, how cancer cells progress through cancerogenesis and give rise to metastasis remains unsettled. Cancerogenesis is a dynamic process that depends on a large number of variables and is regulated at multiple spatial and temporal scales [1]. The model of skin malignant melanoma (MM) progression leading to tumorigenesis begins with a critical step of malignancy corresponding to clonal generation of cells capable of initiating a vertical growth phase [2]. Some of these cells lead to advanced MM competent for metastasis. The most aggressive clones clearly exhibit a growth advantage over other cells [3–9] associated or not with selective advantages including migration [8–11]. This latter attribute of MM cells has rarely been scrutinized in the literature.

A broad range of eukaryotic cells adjust their growth direction according to physical and topographic attributes of the surrounding environment [12–19]. The ability to sense and respond to physical aspects of the organized supporting substrate is indeed an adaptation of a large number of tip growing cells living on and within solid substrates. Such tropism behavior is known variously as thigmotropism, contact-sensing growth, touch-sensitive response, or contour guidance. Typical examples include plant roots that reversibly rotate the root apex or achieve the circumnavigation of obstacles while growing in soil [15, 16]. Fungal growth is similarly under the influence of thigmotropism [17, 18]. Another example is provided by the guided growth of embryo axonal or dendritic processes within solid embryonic or regenerating tissues to achieve the innervation of specific sites [12, 19]. 

It is likely that some specific molecular components play a part in directing cell growth [20]. When the cell contacts an inductive surface, stretching of the cell membrane may occur, during which channels open allowing efflux or influx of specific ions such as Ca^2+^ [21, 22]. As a result the ion concentrations are modulated in the cytoplasm. This process may activate a cascade of events producing a response including the cell shape control (cell tensegrity) [23] and/or thigmotropic differentiation.

Cancerogenesis involves three successive steps, namely, the initiation, promotion, and progression phases [24]. In MM, we frame as an hypothesis that cell migration, thigmotropism, and tensegrity are involved and linked at least in part to tumor progression and cell migration. Cell mobility and migration in combination with cell proliferation are operative in both the primary lesion and in the metastatic spread [4, 7–11, 25–27]. The morphologic plasticity reflected by MM cell tensegrity is probably closely associated as well [28].

## 2. Migration Paths and Contact-Sensing Progression of MM Cells 

MM cells are in essence capable of migration in a variety of directions. At the dermo-epidermal junction, and along hair follicles and sweat ducts, the process contributes to the formation of nests in an accretive pattern [29]. An outward transepidermal migration involves single pagetoid and/or nested MM cells. The invasive pattern of MM involves both the intradermal progression of the primary neoplasm and the micrometastatic spread. At that stage, MM cells may be found scattered in the dermis or adjacent to vessels (angiotropism, extravascular migration) or nerves (neurotropism).

These characteristic features probably result from molecular and microstructural determinants. They are in nature either genetic (gene mutation, deletion, amplification, or translocation) or epigenetic (a heritable change other than in the DNA sequence, generally transcriptional modulation by DNA methylation and/or by chromatin alterations such as histone modification) [11]. Abutted to MM, there must be an adaptive landscape allowing neoplastic cells to adapt to specific microenvironmental selection forces [30]. MM cells must surmount several microenvironmental proliferation barriers. Somatic progression of invasive MM could represent a sequence of phenotypical adaptations to these barriers [14].

During MM progression, different molecular mechanisms are distinctly involved. Changes in cell adhesion molecules are frequently present [31–38]. Secretion of metalloproteinases and their inhibitors is involved as well [8, 39]. In addition, macromolecules of the extracellular matrix (ECM) may be more abundant and possibly produced by MM cells [40–50]. A biomechanical hypothesis was offered to explain the MM radical growth phase involving intercellular/stromal connections [51]. A long wavelength instability is present at the MM front during the early steps of MM invasion [51]. 

The concept of MM thigmotropism encompasses the migration path along the dermo-epidermal and adnexial basement membranes, as well as the extravascular and (peri)neural spreads. The involvement of MM stem cells in the thigmotropic process is unknown. The scattered intraepidermal and intradermal spread do not follow structured anatomical supports and thus do not meet the definition of thigmotropism. The possible intervention of immune cells in directing thigmotropism is unsettled.

In incipient MM, neoplastic cells usually migrate first along the basement membrane with focal nest formation. Such pattern of progression is called atypical melanocytic hyperplasia. Suprabasal tumor-cell spread in a pagetoid pattern is another characteristic feature commonly found in superficial spreading MM. Thus, this neoplasm exhibits a radial growth phase characterized by the presence of severely atypical melanocytes at and above the dermo-epidermal junction from where they are scattered throughout the epidermis [51]. These MM cells commonly have an abundant dusky granular cytoplasm and large pleomorphic and hyperchromatic nuclei. Asymmetry, irregular nesting with cellular discohesion, mitoses, and a prominent band-like lymphocytic infiltrate are present as well. Immunohistochemistry using a panel of markers including S100 protein, HMB45, NKiC3, melan A/MART1, and tyrosinase help in diagnosing MM with confidence [4]. Occasionally MM show positive immunolabeling for the epithelial membrane antigen (EMA), and metastatic MM may show positive staining with polyclonal antibody to carcinoembryonic antigen (CEA).

## 3. MM Progression and Incipient Micrometastasis

The MM cell migration potential and the progression to the fully metastatic cell are complex. Cell adhesion is believed to play a crucial role in two areas. First, there must be a loss of adhesion between MM cells at the primary site to enable individual cells to break free and metastasize. Second, there must be adhesion between some of these MM cells and the vascular or lymphatic endothelium and other structures where the metastasis follows its migration path and is to become established. Loss of a mediator of tumor cell-cell interaction or acquisition of a mediator of tumor cell-endothelial cell interaction may therefore be advantageous to the potentially metastatic MM cell.

Some aspects of MM thigmotropism represent a facet of the progressive extension of the primary neoplasm. However, the perivascular spread releases some MM cell aggregates that are no more in continuity with the main bulk of the primary lesion. They are viewed as micrometastases. Theoretically, such aspect may result from two processes. On the one hand, these microaggregates of MM cells may result from their migration after leaving their primary location. This step requires that the MM cells are able to actively uncouple themselves from neighbouring cells and survive without the regular cell-to-cell interactions. On the other hand, they may represent a residual structure left in place after apoptotic regression of other malignant cells initially forming a continuous chain connected to the primary MM, hence being part of it without representing metastatic deposits. The small groups of clustered surviving cells could then be viewed as the expression of survival privilege perhaps related to the MM stem cell status [7, 9].

## 4. Tropism along Dermoepithelial Junctions

The presence of an increased number of slightly to severely atypical melanocytes arrayed as solitary cells or clustered along the dermo-epidermal junction is an established histologic criteria for the diagnosis of atypical melanocytic hyperplasia or incipient MM. The same process occurs at the outer boundaries of the adnexal epithelia. These descriptive patterns probably result from a peculiar aspect of thigmotropic MM progression abutted to and restricted to the dermoepithelial basement membrane zone. 

The intervention of specific adhesion molecules, particularly integrin subunits, is likely operative. Integrins are cell surface proteins that mediate cell-extracellular matrix adhesion as well as cell-cell adhesion. The transmembrane cell surface integrins are heterodimers composed of two different subunits. The extracellular part forms the ligand-binding site, the intracellular portion is bound to the cytoskeleton. Thus, it is presumed that integrins form a link between the cytoskeleton and ECM components influencing cell tensegrity and migration as well as tumor growth and progression. In particular, MM cells show increased *α*3 *β*1 integrin expression associated with enhanced invasiveness, migration, and metastatic potential.

## 5. Angiotropic Melanoma 

In rare instances, MM cells are recognized inside lymphatic or blood cells. This finding does not, however, predict the development of distant overt metastases. Even more rarely, micrometastases are stuck inside vessel walls. This likely represents a specific metastatic homing. A more common finding is the extravascular migratory path for metastatic MM cells [52–55]. Such migration occurs in close contact with the outer aspect of vessels, inside an amorphous matrix enriched in basement membrane constituents, particularly type IV collagen, laminin tenascin, and fibronectin [50, 56–58]. Such a structure has been called the angiotumoral complex. Adhesion, proliferation, and migration rate of MM cells are increased due to these stromal components. Human endothelial cells represent a boundary during the invasion and extravasation of MM cells. The *α*3 integrin receptor is present on endothelial cells. Adhesion of MM cells to endothelial cells plays an important role in the metastatic process. In culture, a low integrin expression rate was reported to correlate with low adhesion capacity to the extracellular matrix components and with a weak cell migration rate [56].

The extracellular migratory MM metastases may be associated with angiogenic fast-growing MM [46, 59]. Angiotropism is regarded as a predictor for local recurrence and in-transit metastasis [60].

## 6. Neurotropic Melanoma 

The desmoplastic and neurotropic MM patterns often occur together in the same neoplasm [61–63]. The neurotropic features of the neoplasm are regarded as a crucial clue to the diagnosis of desmoplastic MM. Neurotropic thigmotropism is regarded as part of the invasive progression of the primary MM rather than a step in the metastatic process. The neurotropic thigmotropism appears to be associated with specific markers of the neural crest [64, 65].

## 7. Thigmotropism and Metastatic Homing

Metastases may, in some instance, remain limited to a single organ or tissue. Common restricted sites include cutaneous metastases. The MM metastatic homing within the skin is presumably directed, at least in part, by the thigmotropic migration, particularly in the angiotumoral complex, allowing a radial intradermal dissemination of small size metastases. The reason(s) for stopping the thigmotropic migration and initiating the growth of a metastasis remain(s) unsettled. At that stage, metastases have acquired a compelling growth preference over the surrounding tissues. Once cells separate from the primary MM, they must be functionally autonomous. This requires adaptations to maintain continued growth such as the production of autocrine growth factors and other cellular adaptations promoting continued survival apart from the primary MM. This feature could correspond to the conversion of slow-proliferating stem cells to the amplification high rate proliferating cells. A concurrent or subsequent stage is characterized by a possible exuberance of blood vascular angiogenesis.

## 8. Conclusion

Much of the current research in MM therapy is directed at the mechanisms of metastasis, making knowledge of the intricacies of this process pertinent. Metastasis is frequently a subject that conjures up feelings of bewilderment, uncertainty, and confusion. Migration of MM cells along specific skin anatomical structures is a common feature similar to the thigmotropic phenomenon in general biology. It is part of the progression of the primary neoplasm and to the initial steps of micrometastasis production. The molecular mechanisms involved in MM cell migration contribute to a directed thigmotropic progression along specific cutaneous structures. Such features might represent objectives of future targeted therapies. It remains that studies seeking a correlation between adhesion molecule expression and metastatic behaviour have yielded conflicting results.

Obviously, some invasive malignant melanomas are lacking competence for metastasis and the disease-free interval is much prolonged. The microstaging of patients with MM fails in such instance to distinguish groups of patients at low and high risk of metastasis.
